# Characterisation of the Physical Composition and Microbial Community Structure of Biofilms within a Model Full-Scale Drinking Water Distribution System

**DOI:** 10.1371/journal.pone.0115824

**Published:** 2015-02-23

**Authors:** Katherine E. Fish, Richard Collins, Nicola H. Green, Rebecca L. Sharpe, Isabel Douterelo, A. Mark Osborn, Joby B. Boxall

**Affiliations:** 1 Pennine Water Group, Department of Civil and Structural Engineering, The University of Sheffield, Sheffield, United Kingdom; 2 NERC Biomolecular Analysis Facility, Department of Animal and Plant Sciences, Western Bank, Sheffield, United Kingdom; 3 Kroto Research Institute, The University of Sheffield, Sheffield, United Kingdom; 4 Department of the Natural & Built Environment, Sheffield Hallam University, Sheffield, United Kingdom; 5 School of Applied Sciences, RMIT University, PO Box 71 Bundoora, Melbourne, Australia; U. S. Salinity Lab, UNITED STATES

## Abstract

Within drinking water distribution systems (DWDS), microorganisms form multi-species biofilms on internal pipe surfaces. A matrix of extracellular polymeric substances (EPS) is produced by the attached community and provides structure and stability for the biofilm. If the EPS adhesive strength deteriorates or is overcome by external shear forces, biofilm is mobilised into the water potentially leading to degradation of water quality. However, little is known about the EPS within DWDS biofilms or how this is influenced by community composition or environmental parameters, because of the complications in obtaining biofilm samples and the difficulties in analysing EPS. Additionally, although biofilms may contain various microbial groups, research commonly focuses solely upon bacteria. This research applies an EPS analysis method based upon fluorescent confocal laser scanning microscopy (CLSM) in combination with digital image analysis (DIA), to concurrently characterize cells and EPS (carbohydrates and proteins) within drinking water biofilms from a full-scale DWDS experimental pipe loop facility with representative hydraulic conditions. Application of the EPS analysis method, alongside DNA fingerprinting of bacterial, archaeal and fungal communities, was demonstrated for biofilms sampled from different positions around the pipeline, after 28 days growth within the DWDS experimental facility. The volume of EPS was 4.9 times greater than that of the cells within biofilms, with carbohydrates present as the dominant component. Additionally, the greatest proportion of EPS was located above that of the cells. Fungi and archaea were established as important components of the biofilm community, although bacteria were more diverse. Moreover, biofilms from different positions were similar with respect to community structure and the quantity, composition and three-dimensional distribution of cells and EPS, indicating that active colonisation of the pipe wall is an important driver in material accumulation within the DWDS.

## Introduction

Drinking water distribution systems (DWDS) are an essential infrastructure integral to the provision of a safe water supply. DWDS function as microbiological and physico-chemical reactors which interact with drinking water and, in turn, impact upon the quality of the water supplied to customers. Accumulation of microbiological, organic and inorganic material at the pipe wall (and its subsequent release) plays a key role in water quality degradation [1]. Microorganisms have been shown to attach to surfaces and form biofilms comprising cells embedded within a microbially-produced matrix of extracellular polymeric substances (EPS) [2]. The EPS has a complex biochemical composition, comprising predominantly carbohydrates and proteins, although lipids and extracellular DNA (eDNA) have also been identified [3], along with exogenous inorganic or organic substances which may become entrapped within the EPS, for example, iron or manganese [4]. Based upon biofilm research across an array of fields, various roles have been accredited to the EPS matrix, including the provision of the biofilm three-dimensional structure and physical stability [3]. Although research specific to full-scale DWDS pipeline surfaces is limited, it is likely that biofilms are integral to the accumulation of material upon the inner pipe surfaces. Biofilm will be detached if the internal cohesive/adhesive strength of the matrix is weakened, or exceeded, which may occur in DWDS as a result of increased shear stress at the pipe wall due to changes in pipeline hydraulics (e.g. following a burst or seasonal increase in demand). The subsequent mobilisation of microbial cells, EPS and any associated particles, into drinking water will have aesthetic, chemical and biological implications upon water quality.

In view of the crucial role that the EPS matrix plays in biofilm formation and detachment it is essential to better understand the distribution and composition of EPS (and the influence of environmental variation upon these) within drinking water biofilms. However, previous EPS analysis has rarely characterised the matrix of biofilms relevant to full-scale DWDS and microbial drinking water research has often been limited to community characterisation of the microbial cells (whether in the planktonic phase or, occasionally at the pipe wall), particularly with respect to bacteria [5–7]. Bacteria are the most studied microorganisms within the context of DWDS and are the only microorganisms to be monitored internationally with respect to water quality; however, fungi and archaea may also be present within DWDS biofilms. Moreover, often only one of either the biofilm physical structure or community structure is analysed but it is important to integrate the two aspects in order to determine how community composition may influence the development of these EPS characteristics.

It is highly challenging to acquire biofilm samples that are representative of the spatial, temporal and physico-chemical variation of real DWDS as they are live, functioning systems comprised of buried infrastructure. Consequently, much of the current understanding about DWDS biofilms is based upon extrapolations of findings from studies of biofilms in other environments or from bench-top scale experimental models of drinking water systems such as glass coupons within a reactor [8]; biofilms cultured within a flow-through cell [9] or small scale pipe simulations [10]. Whilst such studies have contributed to the development of biofilms analysis techniques, they offer a limited representation of real DWDS, with respect to their physico-chemical, hydrodynamic and microbiological characteristics. In particular, biofilm studies have often been based upon cultured communities seeded with investigator-selected species [9] or developed using an inoculum other than drinking water [11, 12], which is unrepresentative of the complex, multi-species communities that develop naturally in DWDS. Consequently, there is a need for DWDS biofilm research to move beyond these idealised experimental systems, to use engineered systems that more effectively reproduce the DWDS pipeline environment.

There is no single accepted method to visualise and/or quantify the cells and EPS [3, 4, 12], with many protocols being developed to analyse the physical structure of samples from environments substantially different from DWDS. Biofilm matrices may be studied via isolation of the EPS from the cellular fraction, prior to quantification and biochemical characterisation of the carbohydrates [13, 14]. However, the detection limits of these techniques are not necessarily sufficiently sensitive to analyse DWDS biofilms, which (typically) have lower amounts of biomass than biofilms in other environments. Moreover, the results produced from these analytical processes generally vary with the sample origin and methodology applied [4, 13] and it is acknowledged that EPS yield and biochemical evaluation are influenced by the extraction techniques previously employed [4, 13], making comparison between studies difficult. Alternatively, EPS may be analysed via microscopy based approaches, which are more sensitive, the most common of which is fluorescent microscopy, particularly Confocal Laser Scanning Microscopy (CLSM). CLSM facilitates biofilm visualization while enabling the collection of quantitative data but characterisation of EPS is limited by the availability and compatibility of fluorescent stains, which is primarily governed by the stain characteristics and the wavelengths of the lasers available at the CLSM facility. Application of CLSM is often confined to analysis of cells and carbohydrates [15] or identification of carbohydrates and proteins separately using different samples [16]. CLSM with two dual combinations of fluorophores [12] has been used to visualise the carbohydrates (glycoconjugates)\cells within a reactor cultivated biofilm sample, followed by the proteins\cells within another. However, only the carbohydrates and cells were quantified via digital image analysis (DIA) [12]. CLSM has also previously been used to concurrently assess the protein and carbohydrate components of EPS in flocs [11], granules [17] and single-species cultured biofilms [18]. The protocols supplied in these studies provide CLSM image settings and analysis details that have not been developed for use with biofilms from a full-scale DWDS. Moreover, this technique has not yet been applied to concurrently characterise the carbohydrate and protein fractions of the EPS, along with cells, of multi-species biofilms within a full-scale, chlorinated DWDS environment.

This research presents the first concurrent characterisation of the physical composition (cells, carbohydrates and proteins) and microbial (bacterial, archaeal and fungal) community structure analysis of biofilms sampled from around the circumference of pipes comprising a full-scale DWDS experimental system, after 28 days of growth under conditions relevant to real DWDS. In order to evaluate the physical structure it was necessary to first establish an EPS analysis protocol suitable for use with DWDS biofilms. The study presents the protocol used to characterise the physical structure of biofilms, which incorporated visualisation (via fluorescent CLSM) and quantification (DIA) of samples stained with a triple fluorophore combination targeting cells, carbohydrates and proteins, of the same sample.

## Materials and Methods

### DWDS Experimental Facility

**Fig 1:**
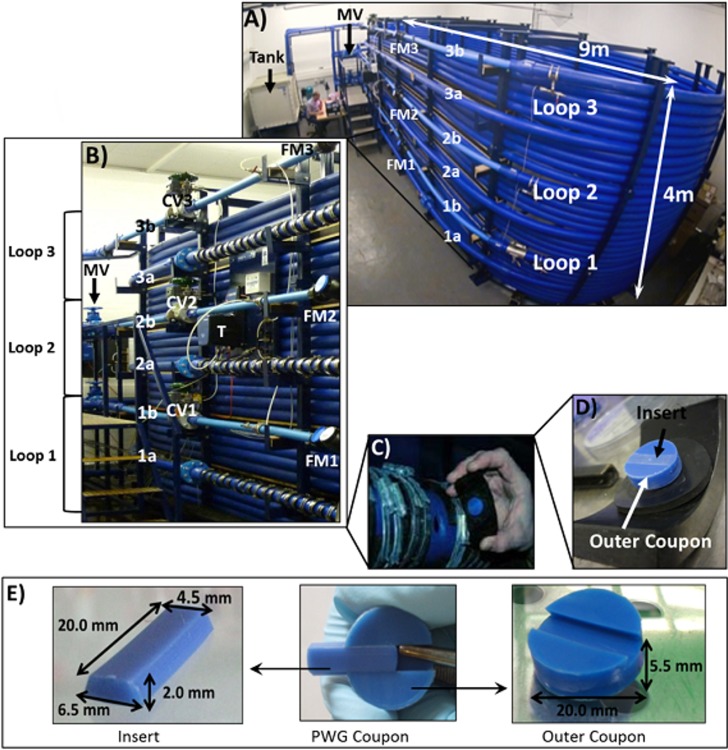
Drinking Water Distribution System (DWDS) simulation pipe facility and Pennine Water Group (PWG) Coupon. A) Test facility, total volume (tank and loops) 4.5 m^3^, tank volume 1.53 m^3^, MV = Manual valves used to separate the three loops, 1a, 2a and 3a indicate the 5^th^ coil of each loop into which PWG coupons were inserted, 1b, 2b and 3b indicate the 50 mm internal diameter pipes containing flow meters (FM) and control valves; B) Detail of loop arrangement, T = turbidity meter, CV = control valve, other annotations as for A); C) Coupons secured in the apertures; D) HDPE PWG coupon and rubber gasket, to ensure a watertight fit; E) PWG coupon components (insert for microscopy and outer coupon for molecular analyses) and dimensions.

Biofilms were developed over 28 days, within a full-scale, temperature controlled DWDS simulation pipe system (Fig. 1), which replicates the hydraulics, water chemistry, microbiology and water-pipe wall exchange mechanisms of DWDS, whilst enabling laboratory level control of environmental variables, replication and biofilm sampling. In brief, the facility consisted of three 203 m long, high-density polyethylene (HDPE) pipe loops, 79.3 mm in internal diameter. The mid (5^th^) coil of each loop had apertures into which HDPE Pennine Water Group (PWG) coupons [19] were inserted, providing a removable surface for biofilm growth, following the internal pipe curvature and thus limiting the distortion of boundary layer hydraulics. PWG coupons comprise an outer section used for harvesting biofilm for DNA-based analyses and an insert designed for microscopy analyses [19]. Coupons (and apertures) were arranged around the pipe in the sequence: invert (base of pipe), middle and crown (top of pipe), repeated nine times in total along the pipe length.

Local drinking water (from an upland peat runoff surface water source) was supplied to the facility directly from a cast iron trunk main, trickle fed into an enclosed reservoir tank and recirculated around the system with a 24 hour retention time, which preserved baseline water quality parameters. Water quality parameters were monitored throughout the experiment (triplicate samples taken weekly, n = 15) and complied with UK standards (S1 Table).

PWG coupons were sonicated with a 2% (w/v) sodium dodecyl sulphate (SDS) solution for 45 minutes and sterile deionised water for 15 minutes, autoclaved [20, 21] and inserted into the facility. Before use, the pipe system was disinfected with a 20 mgl^-1^ sodium hypochlorite solution (< 16% free chlorine; Rodolite-H, RODOL Ltd, Liverpool, UK) re-circulated within the system for 24 hours, at the maximum achievable flow rate (4.5 ls^-1^). Subsequently, the system was flushed repeatedly (4.5 ls^-1^) with fresh water, until chlorine levels decreased to those of the inlet water.

### Growth Conditions and Sampling of Biofilm

Biofilms were developed naturally (i.e. no cultures or inoculations were added) for 28 days, at 16°C (± 1°C; controlled and monitored by a cooling unit system), which is representative of water temperatures during summer in the UK. To develop the staining methodology and DIA method, biofilms were established under steady state flows in a pilot run of the experimental system and coupons were sampled at Day 28 (n = 3 per fluorophore, with one coupon per loop). Subsequently, biofilms were developed for physical and microbial community analysis under a steady state flow regime of 0.4 ls^-1^ (shear stress 0.30 Nm^-2^, Reynolds number ∼5800). This flow rate is the average recorded in 75–100 mm diameter pipes within UK DWDS [22]. Biofilms were sampled at Day 0 (between 60 and 90 minutes) and Day 28 (n = 9, three per loop, one from each position).

All biofilm samples were collected without draining the system, limiting the impact of sampling upon biofilm accumulation, and replacement sterile coupons were immediately inserted into the pipe. Inserts and outer coupons were separated aseptically and used for microscopy (physical structure) and community structure analysis, respectively. It was not feasible to analyse all nine inserts, therefore a subset of five replicates was selected which comprised: a coupon from the crown, middle and invert of loop 2, and a middle coupon from loops 1 and 3. Inserts (n = 5) were fixed in 5% formaldehyde for 48 hours at 4°C and rinsed three times (1 minute washes) in phosphate buffer solution (PBS) containing: 2 mM Na_3_PO_4_, 4 mM NaH_2_PO_4_, 9 mM NaCl, 1 mM KCl, then stored in PBS at 4°C [23] prior to staining and imaging. Biofilm was removed from all outer coupons (n = 9) by brushing (30 horizontal/vertical strokes) into 30 ml sterile PBS, the resulting suspension was filtered through a 47 mm diameter, 0.22 μm pore nitrocellulose membrane (Millipore, MA, USA) using a Microsart membrane filtration unit (Sartorius, UK). Filters were stored at -80°C prior to analysis of microbial community structures via DNA based fingerprinting approaches.

### Physical Structure: Biofilm Staining and Fluorophore Combinations

Multiple fluorophores were evaluated for their suitability for use with DWDS biofilm samples, particularly their ability to be resolved from any autofluorescence of the HDPE insert surface, unstained DWDS biofilm and any signal(s) from other fluorophores. To assess this, fluorophores (Table 1) targeting different biofilm components (nucleic acids, glycoconjugates or proteins/amines—hereafter referred to as cells, carbohydrates and proteins) were applied singularly, and subsequently, in paired or triple combinations to DWDS biofilms. Fluorophores were selected on the basis of their previous application to microbial aggregates [12, 17], their suitability for CLSM analysis and their distinct excitation/emission spectra Although DAPI is frequently used to visualise cells via epifluorescent microscopy [19], this fluorophore was not analysed as no suitable single photon laser (405 nm) was available.

**Table 1:** Fluorophores (fluorescent stains) evaluated for their use in visualising cells, proteins or carbohydrates within drinking water biofilms.

Fluorophore	Target Component	Concentration Used	Incubation Time (minutes)^B^	Ex.^C^ (nm)	Em.^D^ (nm)	Lambda Range^E^ (nm)	Reference
SYTO 9	Cells (DNA)	1 μM	15	488	498	500.9–704.2	[53]
BacLight Live-Dead (SYTO 9/ Propidium Iodide)	Cells (DNA)	As supplied	30	488	498/635	500.9–700.4/ 650.7–704.2	[54]
SYTO 63	Cells (DNA and RNA)	20 μM	30	633	673	650.7–704.2	[17]
SYPRO Orange	Proteins	0.2 μl ml^-1^ ^**F**^	15	488	570	500.9–704.2	[12, 53]
Fluorescein-5-isothiocyanate (FITC)^A^	Proteins (amines and amino-sugars)	0.1 mg ml^-1^	60	488	520	500.9–704.2	[17]
Concanavalin A tetramethylrhodamine (Con A Rho)	Carbohydrates (α-mannopyranosyl and α-glucopyranosyl sugars)	0.1mg ml^-1^	30	543	580	554.4–704.2	[17, 18, 36]
Alexa Fluor 647	Carbohydrates (α-mannopyranosyl and α-glucopyranosyl sugars	0.1 mg ml^-1^	30	633	668	650.7–704.2	[54]

^A^ Before staining, samples were pre-washed in 0.1 M sodium bicarbonate, to retain the amines in non-protonated form [36].

^B^ Protected from light.

^C^ Excitation wavelength used in this study, 488 nm-argon laser, 543 nm and 633 nm-helium/neon lasers.

^D^ Emission maxima according to supplier(s)

^E^ Lambda detection range over which emitted light was collected, the lower boundary was selected to avoid collecting the excitation laser-flare but ensure the emission peak wavelength was included where possible

^F^ SYPRO Orange molecular weight/molar concentration not provided by supplier, stock stated as 5000× concentration, diluted using 7.5% acetic acid.

Fixed biofilm samples (n = 3 per fluorophore or combination thereof) and control samples (sterile inserts, n = 3) were stained using a 300 μl volume of the appropriate fluorophore solution(s) and incubated at room temperature. Where combinations were tested, fluorophore application was conducted in two or three stages, following the sequence: cell—protein—carbohydrate, with three intermediate washing stages (1 minute) using sterile PBS, to ensure that lectins were not stained by the protein fluorophore. Standards on glass slides (n = 3) were tested to confirm fluorophore binding specificity. Samples were air dried (10 minutes) and stored, in the dark, at 4°C prior to CLSM imaging. It is recognised that air drying may reduce the biofilm volume as desiccation decreases the cytoplasmic volume of cells and the EPS may also appear thinner, due to it being highly hydrated [3]. Consequently, it is preferable to analyse unfixed, hydrated samples but the extensive sampling schedule did not allow for this. Nonetheless, air drying was minimal and occurred post-fixing so the position of the components was preserved; all the samples were treated identically, therefore results remain comparable.

### Physical Structure: Empirical Testing of Fluorophore Combinations via CLSM

**Lambda-Z-Stack Imaging**. To analyse cells and EPS throughout the biofilm a LSM510 meta upright confocal microscope and LSM510 software (Zeiss, Germany), within the Kroto Imaging Facility (The University of Sheffield, UK) and three single photon lasers (488 nm—argon laser, 543 nm helium/neon laser, 633 nm helium/neon laser) were used to produce lambda-Z-stacks. In brief, a series of XY lambda images (optical slice 4.7 μm) were taken at regular intervals (2.35 μm; see S1 Fig.). The Z-stack limits were investigator-selected, therefore, the stack size varied between fields of view (FOV), due to differences in biofilm coverage and the topography of the plastic (S1 Fig.). Samples were secured within a specially designed holder and imaged using a: ×20 EC Plan Neofluar objective (0.5 NA), 420 μm^2^ image area, 3.94 μs pixel dwell time and 832 × 832 pixels frame size, selected to facilitate detection of a single cell (1 pixel = 0.51 μm). Multispectral data stacks (S1 Fig.) with 10.7 nm resolution were obtained, with the settings optimised for each stain individually (Table 1). To minimise focal drift, room and sample temperatures were allowed to stabilise for 30 minutes prior to imaging and the room temperature was monitored throughout (average 24.6°C ± 1°C) using an EL-USB1 temperature logger (Lescar Ltd., Sailsbury, UK).

**Fig 2:**
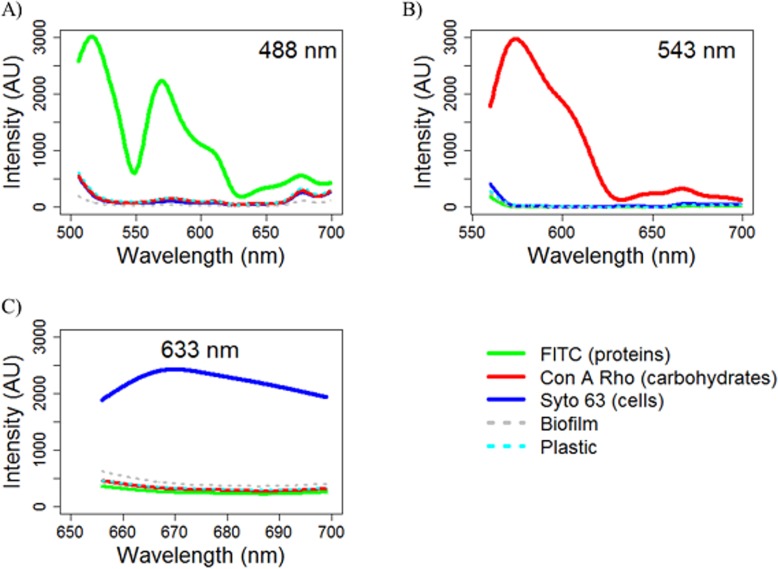
Emission spectra (fingerprints) of the triple stain combination targeting cells (Syto 63), carbohydrates (Con A Rho) and proteins (FITC), at each excitation wavelength. Note that plastic/biofilm autofluorescence is also shown and that the whole spectrum in each case was used in linear unmixing. A) Emission at 488 nm excitation, image settings for FITC; B) Emission at 543 nm excitation, image settings for Con A Rho; C) Emission at 633 nm excitation, image settings for SYTO 63.

**Autofluorescence of Controls and Emission Fingerprints**. Unstained controls were observed to auto-fluoresce when exposed to each excitation wavelength (488 nm, 543 nm and 633 nm; see S2 Fig.) but no staining of the plastic was observed. Imaging in lambda mode enables autofluorescence to be removed from images of stained biofilms via unmixing—if emission fingerprints are known for each of the components within the image. To determine the emission fingerprint of the autofluorescence of the biomass and/or the PWG insert and individual fluorophores, unstained sterile inserts (n = 3), unstained biofilms (n = 3) and single stained biofilms (n = 3, per fluorophore) were imaged at seven FOV. All samples were imaged under the settings optimised for each fluorophore. For each fluorophore (or control), 21 emission spectra (one per FOV, for each sample) were analysed graphically using the software R v2.15 [24] to assess their similarity. No difference was found between replicate spectra in any instance, therefore the characteristic emission spectrum was assigned (Fig. 2) by plotting all the spectra and selecting the median spectrum (for example, in S2 Fig., ROI 1 would be assigned as the emission fingerprint).

**Fluorophore Combinations**. Lambda-Z-stacks were subsequently generated for dual or triple stained samples (n = 3 for each combination thereof, 7 FOV). Linear unmixing was applied, using the pre-determined emission fingerprints, to establish the combinations which could (or could not) be separated (S1 Table). Compatible fluorophore pairs were subsequently established and based upon these, the triple combination of SYTO 63\FITC\Con A Rho, was tested and confirmed as suitable for use in staining the cells/proteins/carbohydrates within the DWDS biofilm samples. There was no difference in the standard deviations of data based on the analysis of seven FOV compared to a randomly selected subset of five FOV (Wilcoxon tests, W ≥ 9, p > 0.05 in all instances). Consequently, a greater number of replicates (n = 5 rather than n = 3) were analysed at fewer FOV (5 rather than 7), resulting in greater replication overall (n = 25 rather than n = 21).

**Physical Structure: Triple-stained Biofilms**. The triple-stain combination was applied to Day 0 and Day 28 biofilms (n = 5, FOV = 5 per replicate), which were imaged in lambda mode (S2 Table) and unmixed prior to DIA. Three Z-stacks, each optimised for one of the three fluorophores, were obtained for each FOV and the data relating to the fluorescence of the targeted stain was analysed.

**Physical Structure: Digital Image Analysis.** DIA was applied to unmixed Z-stacks of triple stained biofilm samples in order to: reduce the image noise (median filtering); generate a threshold; calculate various quantification parameters; overlay, and render unmixed images; and analyse the resultant data. Details of the DIA are presented in the following sections; the authors used a combination of the freely available programs Python v2.7.2 (http://www.python.org) and R v2.15 [24] for this analysis. All of the quantification measures were calculated using unmixed, median filtered and thresholded images.

**Median Filtering**. Images taken using the far red spectra generally yield a considerable amount of random background noise [17]. Therefore, a 3 × 3 median filter was applied to all unmixed Z-stack image series before thresholding, quantification or visualization was carried out, to maintain a fine level of detail [25].

**Thresholds and Area Coverage.** Not all stain associated pixels are an exact match to the fluorophore signal so it is necessary to establish the level at which a pixel associated with the stained component is deemed stain-positive or stain-negative. In this study possible threshold values ranged from 1 to 4095, where a threshold value of 1 would label all of the pixels associated with a particular fluorophore as stain positive (4095 would label none).

The area covered by the stained component was calculated for each image in a Z-stack by dividing the number of stain associated pixels (at a given threshold) by the total number of pixels in the image (832 × 832). Area coverage (expressed as a fraction) was the principal quantified parameter and a prerequisite for all subsequent DIA; therefore these data were used to inform the selection of a threshold value. In brief, area coverage data were generated for all possible thresholds and normalised by the maximum value in each instance. The data were analysed graphically and statistically [Kruskal Wallis Test; 24] to determine the range of thresholds between which there was no difference in the normalised data. The median was chosen as the threshold value for each fluorophore, namely: SYTO 63 (cells) threshold 2401, Con A Rho (carbohydrates) threshold 1701, and FITC (proteins) threshold 1701. The pitfalls of thresholding are well acknowledged [26] but selecting a threshold in this way removes any investigator-bias and individual FOV influences, which standardises the process, facilitating comparison between datasets.

**Volume and Composition.** In order to quantify and characterise the composition of the drinking water biofilms, the volume (μm^3^) of each of the stained components was generated using the equation:

$$Volume=\frac{dZ}{2}\times ImageArea_{Total}\times \sum_{i=a}^{b}AreaCoverageFraction$$ Equation 1

Where optical slice depth (*dZ*) is 4.7 μm, *ImageArea*_*Total*_ is 420 μm^2^, *a* is the first slice of the Z-stack, *b* is the last slice and *AreaCoverageFraction* is the proportion of the slice covered by the particular stain for which volume is being calculated. The volume was relative to the minimum detection threshold applied previously, but will be referred to solely as “volume”. Subsequently, the volumes of EPS (protein plus carbohydrate) and total biofilm (EPS plus cells) were calculated and the biofilm composition was evaluated by generating ratios of carbohydrate-to-protein, carbohydrate-to-cells and protein-to-cells.

**Distribution and Spread (Thickness).** It was desirable to compare the area covered by cells, carbohydrates or proteins throughout the biofilm. However, this was not possible because the stack size differed with each FOV. Therefore, FOV were normalised by labelling the slice with maximum coverage of cells as slice “0” and numbering slices above and below consecutively. The cells were chosen as the component to align to because they produce the EPS and, in physical extraction EPS analysis, the EPS quantity is commonly related back to cell abundance.

**Fig 3:**
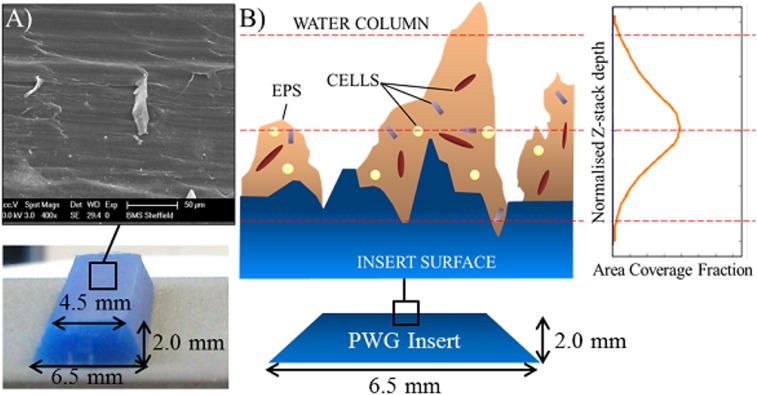
Explanation for the area distribution shape given the uneven surface of the insert. A) Scanning electron microscope image showing the uneven surface of a sterile insert; B) Schematic of the uneven insert surface with biofilm growth, HDPE roughness height is commonly taken in modelling to be 20 μm, 80 μm was the greatest depth measured in Day 28 biofilms. Broken red lines indicate parts of an example area distribution curve and the corresponding cut through position of the biofilm.

Normalised Z-stack depth was calculated by multiplying the aligned slice number by the thickness (4.7 μm) of each slice, this was plotted against area coverage, producing area distribution plots, a schematic example of which is shown in S1 Fig. Note that the y-axis of the area distribution plots runs from positive values which correspond to the biofilm-plastic interface (i.e. the bottom of the biofilm) to negative values which correspond to the biofilm-bulk water interface (i.e. the top of the biofilm). It should be recognised that the low area fractions at the borders of the biofilm-plastic interface are considered to be due to the uneven surface of the plastic insert (Fig. 3).

Area distribution plots were compared visually and the aligned slice number with the maximum area coverage recorded for each component—termed the “peak location”. As the Z-stacks were aligned using the cells, their peak location was always zero. However, the peak location of the carbohydrates and proteins could occur on any slice.

Biofilm thickness is often calculated based on the assumption that the biofilm spans the whole Z-stack and that no biofilm exists outside the Z-stack range. In reality, the first and last slides of a Z-stack tend to contain little material. Consequently, biofilm thickness is likely to be overestimated if calculated in this way, unless a second threshold is applied. To avoid this, a parameter termed “spread” (μm) was defined, as a proxy for thickness:

$$Spread=\frac{Volume}{AreaCoveragePeak\times ImageArea}$$ Equation 2

This enabled the distinction between differently shaped area distribution plots (S1 Fig.) as broader distributions have higher spread values.

**Statistical Analysis.** The volume, spread and peak location data were not normally distributed (Shaprio Wilks Test, p<0.05), therefore, all quantification was based upon the range and median; significance was tested with the Wilcoxon two-sample test (for which W and p values are presented).

**Biofilm Visualisation.** The median filtered, thresholded data for each slice within the three Z-stacks (cells, carbohydrates and proteins) of a single FOV were overlaid. A three-dimensional projection of each Z-stack was also produced, using Para View (v.3.14.0; http://www.paraview.org), the colour of each stain was set to 50% opacity to enable each component to be visualised more easily where co-localisation occurred.

### Microbial Community Structure Analysis

**DNA Extraction**. DNA was extracted from the nitrocellulose filters from Day 0 and Day 28 samples (n = 9) and negative controls (n = 3), using the proteinase K chemical lysis method [27]. Briefly, filters were incubated with 720 μl of SET buffer (0.75 M sucrose, 40 mM EDTA, 50 mM Tris-HCl, pH 9) and 81 μl of lysozyme (10 mgml^-1^), at 37°C for 30 minutes with rotation in a hybridization oven (Thermo Scientific, UK). A 90 μl volume of 10% SDS (w/v) and 25 μl volume of proteinase K (20 mgml^-1^; Applied Biosystems, UK) were added, prior to incubation for a further 2 hours (with rotation) at 55°C. The resultant lysate was added to 137 μl of 5 M NaCl and 115 μl of 1% CTAB (hexadecyltmethyl ammonium bromide)/ 0.7 M NaCl solution and incubated (with rotation) at 65°C for 30 minutes. The upper aqueous layer was transferred to a clean tube and an equal volume of chloroform/isoamyl alcohol (24:1) added, prior to being centrifuged for 5 minutes—note that all centrifugation was at 12,000 xg. DNA was precipitated with 815 μl of 100% isopropanol at -20°C, for 12–14 hours, before centrifugation for 30 minutes. The DNA pellet was washed twice with 70% ethanol, dried and eluted in 30 μl of sterile nuclease free water (Ambion, Warrington, UK).

**PCR Amplification**. DNA extracts were used as templates for three different PCR amplifications (carried out on an AB 2720 thermal cycler (Applied Biosystems, Warrington, UK) for specific gene fragments from bacteria (16S rRNA), archaea (16S rRNA) and fungi (ITS region). Each PCR was carried out using the conditions and primer pairs shown in Table 2. The forward primer was labelled with 6’ carboxyfluorescein dye (6-FAM) to enable detection via fluorescent fingerprint analysis. Bacterial PCR consisted of 12.5 μl of Sigma ReadyMix *Taq* solution (Sigma Aldrich, UK), 0.4 μM of each oligonucleotide primer and 2 μl of DNA template, in a final volume of 25 μl. Archaeal PCR consisted of 1 × reaction buffer, 10 μl of Q-solution, 100 μM dNTPs, 0.15 μM of each oligonucleotide primer, 1.25 U of *Taq* DNA polymerase (Qiagen, Crawley, UK) and 1–2 μl of DNA template, in a final volume of 50 μl. Fungal PCR contained 1 × reaction buffer, 1 mM MgCl_2_, 50 μM dNTPs, 0.2 μM of each oligonucleotide primer, 2.5 U of *Taq* DNA polymerase (Qiagen, Crawley, UK) and 1–2 μl of DNA template, in a final volume of 50 μl. PCR products were confirmed by agarose gel electrophoresis and purified using the QIAquick PCR purification kit (Qiagen, Crawley, UK).

**Table 2:** Oligonucleotide primer pairs used to amplify 16S rRNA genes and ITS regions, PCR cycling conditions used in each case are indicated.

Microorganism(amplified gene)	Amplicon Size (nt)	Oligonucleotide Primers ^A^		PCR Cycle Conditions		Ref.^C^
		Primer Pair	Primer Sequence (5’– 3’)	Temperature (°C) and Duration (minutes:seconds)	Cycles^B^	
Bacteria (16S rRNA)	∼455	FAM-63F and 518R	6-FAM-CAGGCCTAACACATGCAAGTC and CGTATTACCGCGGCTGCTCG	Initial denaturation 94°C, 2:00; Denaturation 94°C, 0:30; Annealing 55°C, 0:30; Elongation 72°C, 0:45; Elongation stop 72°C, 10:00	×30	[55]
Archaea (16S rRNA)	∼849	FAM-Arch109F and Arch958R	6-FAM-ACKGCTCAGTAACACGT and YCCGGCGTTGAMTCCAATT	Initial denaturation 95°C, 0:45; Denaturation 95°C, 0:45; Annealing 55°C, 1:00; Elongation 72°C, 1:30; Elongation stop 72°C, 10:00	×35	[56]
Fungi (ITS region)	∼200–1000	FAM-ITS1F and ITS4	6-FAM—CTTGGTCATTTAGAGGAAGTAA and TCCTCCGCTTATTGATATGC	Initial denaturation 95°C, 5:00; Denaturation 95°C, 0:30; Annealing 55°C, 0:30; Elongation 72°C, 1:00; Elongation stop 72°C, 10:00	×35	[46]

^A^ All primers were sourced from Sigma, UK

^B^ Number of cycles of the denaturation, annealing and elongation steps

^C^ References that used these primer combinations.

**Community Fingerprinting**. Two fingerprinting techniques were applied; bacterial and archaeal communities were analysed using terminal-restriction fragment length polymorphism (T-RFLP) [28, 29], fungal communities were analysed using Automated Ribosomal Intergenic Spacer Analysis (ARISA) [30]. Purified bacterial and archaea 16S rRNA amplicons were digested separately with 10 U AluI (Roche, Germany) in a total volume of 15 μl, at 37°C for 2 hours.

Aliquots (5 μl) of the digested bacteria or archaea PCR products, or the purified undigested fungal PCR products were desalted via precipitation with 0.25 μl of glycogen (20 mg ml^-1^; Fermentas Thermo Scientific, Loughborough, UK) and 0.53 μl of sodium acetate (3 M, pH 5.2) in 70% ethanol (centrifuged at 4°C, for 20 minutes). The pellet was washed twice in 70% ethanol and re-suspended in 5 μl of nuclease free water (Ambion, Warrignton, UK).

Desalted bacterial or archaeal digests were denatured with hi-di formamide containing 0.5% GeneScan 500 ROX internal size standard (Applied Biosystems, Warrington, UK), in a total volume of 10 μl. Desalted fungal amplicons were combined with hi-di formamide containing 0.5% ROX GeneScan 2500 internal size standard (Applied Biosystems, Warrington, UK), in a total volume of 10 μl. Samples (and internal size standard controls) were denatured at 94°C for 3 minutes, cooled on ice and electrophoresed using an ABI 3730 PRISM capillary DNA analyser using POP7 (denaturing) polymer (Applied Biosystems, Warrington, UK) at injection times of 5, 10 or 20 seconds, with an initial injection voltage of 2 kV, followed by ten incremental voltage increases (each 20 seconds) to a final run voltage of 15 kV. The total electrophoresis run was 20 minutes for T-RFLP and an hour for ARISA.

**Community Composition Data Analysis**. The resulting T-RFLP and ARISA electropherograms were analysed via GeneMapper v3.7 (Applied Biosystems) to establish the size of each T-RF (50–500 nucleotides) or ARISA fragment (94–827 nucleotides), as estimated using the Local Southern method (in comparison with the internal size standards). To remove noise, only T-RF/ARISA peak heights greater than 50 fluorescent units were analysed. Fingerprint profiles were expressed in terms of the peak area and size of each T-RF/ARISA fragment and aligned using T-Align [31], with a confidence interval of 0.5 nt. Aligned data was normalised to exclude terminal-restriction fragments (T-RFs) or ARISA fragments that contributed <0.5% to the community profile. Normalised datasets were square root-transformed and Bray-Curtis similarity matrices were generated [32] to compare the relative abundance of T-RFs/ARISA fragments in each profile. Multivariate analysis was carried out using PRIMER-E v6.1 [32]; specifically, hierarchical clustering with similarity profile assessment (SIMPROF; 20,000 permutations), analyses of similarity (ANOSIM) tests (for which global R and p values are presented) and similarity percentage calculations (SIMPER). Three ecological indices, relative to the community profile [33], were calculated: richness (i.e. the total number of T-RFs/ARISA fragments per sample), Shannon’s diversity index [34] and Pielou’s evenness index [35]. T-tests were performed to assess significance, for which degrees of freedom (df) and p values are presented.

## Results

### Characterizing Biofilm Physical Structure

**Position Effects.** Analysis of the position of the coupon within the loop or the loop number showed no significant difference with respect to the volume (W>79.5, p>0.0590 and <0.9834) or spread (W>96.5, p>0.1644 and <0.7873) of cells, carbohydrates or proteins. Similarly, there was no significant difference in the peak location of carbohydrates (W≤113.0, p≤0.2161) or proteins (W≤88.5, p≤0.0856), indicating that there was no influence of position upon biofilm physical structure. Consequently, all subsequent analysis of biofilm images, from a given time point, used the data set as a whole (n = 25).

**Fig 4:**
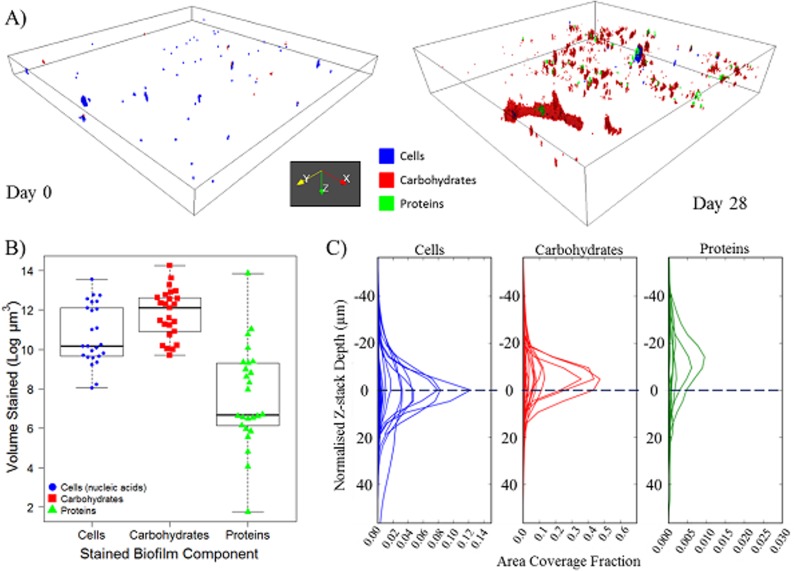
An example of the arrangement, volume and distribution of cells, carbohydrates and proteins of drinking water biofilms. A) 3D projection of example Day 0 and Day 28 biofilms, plotting region shown is 420 μm × 420 μm × 30.6 μm and 420 μm × 420 μm × 94.4 μm, respectively; B) Volume (log) of biofilm components at Day 28, relative to the thresholds used in digital image analysis, each data point (n = 25) represents a FOV, box and whisker plots show the range, interquartile range and median—indicated by the solid black line; C) Day 28 area distribution plot, each line (n = 25) indicates a FOV, note the different x-axis scales between components. Area coverage fraction refers to the proportion of each XY image of the Z-stack covered by the particular component, blue dashed line at “0” indicates the cell peak location; peak location is the aligned slice number at which the maximum area fraction occurs. Area fractions for carbohydrates and proteins are plotted relative to cells (see Materials and Methods for details).

**Visualisation and Characterisation of DWDS Biofilms after 28 Days of Growth.** After 28 days of growth, biofilms were heterogenic in their coverage and morphology (e.g. Fig. 4A). A FOV generally contained all three stained components but no complete co-localisation was observed; the extent of carbohydrate coverage in contrast to that of proteins was illustrated, along with areas where either only cells or EPS were present. The median total biofilm volume at Day 28 was 252325 μm^3^ (per 420 μm^2^ FOV), of which carbohydrates were the dominant component (Fig. 4; Table 3) occurring at a significantly greater volume than either cells (W = 179.0, p = 0.0090), or proteins (W = 40.0, p<0.0001). Proteins occurred at a significantly lower volume than the cells (W = 543.0, p<0.0001) and were consistently the least abundant biofilm component (Fig. 4; Table 3). The range in volume of each of the components (Table 3) indicates the substantial heterogeneity in biofilm coverage and supports the choice of analysing a greater number of sample replicates.

**Table 3:** Volumes and ratios of the stained cells, carbohydrates and proteins of drinking water biofilms.

Component	Range (Minimum—Maximum)		Median	
Volume (μm^3^)	Day 0	Day 28	Day 0	Day 28
Cells	66–137860	3119–769191	35543	26099
Carbohydrates	1–189129	16257–1537181	9874	180802
Proteins	0–1387	6–1027266	177	800
EPS ^A^	29–189132	16518–1545084	11059	184850
Total Biofilm ^B^	321–261128	31268–2085836	50745	252325
**Volume Ratios** ^**C**^	**Day 0**	**Day 28**	**Day 0**	**Day 28**
EPS: Cells	0.0–112.9	0.1–152.7	0.4	4.9
Carbohydrates: Cells	0.0–112.3	0.1–151.9	0.3	4.8
Proteins: Cells	0.0–0.5	0.0–2.5	0.0	0.1
Carbohydrates: Proteins	0.0–80480.5	0.3–36889.2	46.8	62.3

^A^ EPS = Carbohydrates + Proteins, before averaging

^B^ Total Biofilm = Cells + Carbohydrates + Proteins, before averaging. In both instances, data presented are therefore the minimum, maximum and median of the sums

^C^ The first component is divided by the second; a value > 1 indicates a greater volume of the first component; a value <1 indicates a greater volume of the second component.

Although some biofilm material was visualised at Day 0 (Fig. 4A), this was uneven and thinly dispersed, compared to Day 28 biofilms. As expected, the total volume of biofilm at Day 0 (Table 3) was significantly lower than that at Day 28 (W = 77, p<0.0001). In contrast to Day 28 biofilms, Day 0 material was predominantly comprised of cells with little or no EPS detected (Table 3); proteins were particularly sparse or absent completely at Day 0 (e.g. Fig. 4A). Each of the three stained components had a greater volume at Day 28 compared to Day 0 but, despite a 47-fold increase in the minimum cell volume and a five-fold increase in the maximum (Table 3), the only statistically significant changes were a greater presence of carbohydrates (W = 64, p<0.0001) and an increase in proteins (W = 123, p = 0.0002), i.e. a greater presence of EPS. Regardless of the greater EPS volume at Day 28 compared to Day 0, the EPS composition ratio (carbohydrate-to-protein; Table 3) did not differ significantly (W = 249.0, p = 0.5900). Conversely, the carbohydrate-to-cell ratio increased significantly from 0.31 at Day 0 to 4.80 at Day 28 (W = 151.0, p = 0.0014).

All stained components within a Day 28 biofilm occurred throughout a similar normalised Z-stack depth (Fig. 4C), i.e. they were present on a similar number of Z-stack slices. This observation was supported by the similarities (W≥318, p≥0.2389) in spread values for cells (median = 25.70 μm), carbohydrates (median = 24.91 μm) and proteins (median = 26.30 μm). Although the three components were present across similar depths of biofilm, the areas which they covered at each depth varied considerably, with the protein area coverage fraction generally one or two orders of magnitude lower than cells or carbohydrates, respectively (Fig. 4C). Commonly, the peak area fractions of the carbohydrates and proteins occurred above that of the cells (i.e. closer to the bulk water). This can be seen in Fig. 4C for Day 28 biofilms, where the peak of the carbohydrate or protein area distributions occurs above the peak of the cells by an average of 1 or 3 slices, respectively. This trend was also seen at Day 0, with no significant difference in the peak location of the EPS molecules (in relation to the peak location of the cells) between Day 0 and Day 28 biofilms (carbohydrate, W = 233.5, p = 0.1459; protein, W = 323.0, p = 0.5677).

### Biofilm Microbial Community Structure

**Position Effects.** The position or loop from which coupons were sampled did not alter the community structure with respect to bacteria (ANOSIM: global R<0.00, p≥0.626), archaea (ANOSIM: global R<0.00, p≥0.828) or fungi (ANOSIM: global R≤0.12, p≥0.152), in either Day 28 or Day 0 biofilms. Therefore, there was no requirement to account for differences in coupon location in subsequent community structure analyses.

**Characterisation of DWDS Biofilms after 28 Days of Growth.** Bacteria, archaea and fungi were detected in all nine biofilm samples from Day 28 but the archaeal communities had a reduced relative richness compared to the bacterial and fungal communities (Table 4). According to a search of known and “uncultured” archaea in the Ribosomal Database Project (rdp.cme.msu.edu), a total of 307 T-RFs ranging from 2 nt to 500 nt in size, are possible with the primer/enzyme combination used. Therefore low archaeal diversity was not due to a conserved region across different archaea species but a true reflection of the small number of taxa identified within Day 28 drinking water biofilms.

**Table 4:** Ecological diversity indices and similarity values of the bacterial, archaeal and fungal communities from drinking water biofilms sampled at Day 0 and Day 28.

Sample Point	Microbial fingerprint	Relative Richness ^B^	Relative Evenness ^D^	Relative Diversity ^E^	Similarity between replicates (%) ^F^
		Mean (St.Dev.)^C^	Mean (St.Dev.)^C^	Mean (St.Dev.)^C^	
Day 0	Bacteria ^A^	3 & 5	0.89 & 0.95	1.04 & 1.43	36.30
	Archaea	8 (2.39)	0.92 (0.02)	1.90 (0.28)	66.54
	Fungi	11 (5.76)	0.91 (0.03)	2.05 (0.61)	9.75
Day 28	Bacteria	37 (4.59)	0.97 (0.01)	3.48 (0.13)	52.83
	Archaea	11 (1.20)	0.89 (0.01)	2.15 (0.11)	85.80
	Fungi	24 (13.51)	0.91 (0.06)	2.77 (0.67)	25.38

^A^ n = 2 therefore no average could be calculated, both values are presented

^B^ Number of T-RFs

^C^ Standard deviation

^D^ Pielou's Index

^E^ Shannon's Index

^F^ SIMPER analysis.

Fewer microbial PCR products were amplified from Day 0 biofilms (2/9 bacterial, 5/9 archaeal and 5/9 fungal PCR products) compared to Day 28 biofilms (9/9 in all instances). As bacteria were only detected in 2 samples from Day 0, it was not appropriate to calculate an average of the ecological indices or undertake statistical comparison to Day 28 data, instead the minimum and maximum values were compared and the trends reported. Overall, microbial communities were more complex at Day 28 than at Day 0, with significantly greater relative taxon richness for archaea (df = 5.15, p = 0.0441) and fungi (df = 11.61, p = 0.0353) (Table 4). However, no significant difference in relative diversity was detected in any of the biofilm microbial communities (df≥4.68, p≥0.1125 and relative evenness did not differ in the bacterial or fungal communities (df = 11.75, p = 0.8039). Conversely, archaeal communities at Day 28 had a significantly lower relative evenness value than those at Day 0 (df = 6.50, p = 0.0327), indicating the dominance of certain T-RFs at Day 28.

**Fig 5:**
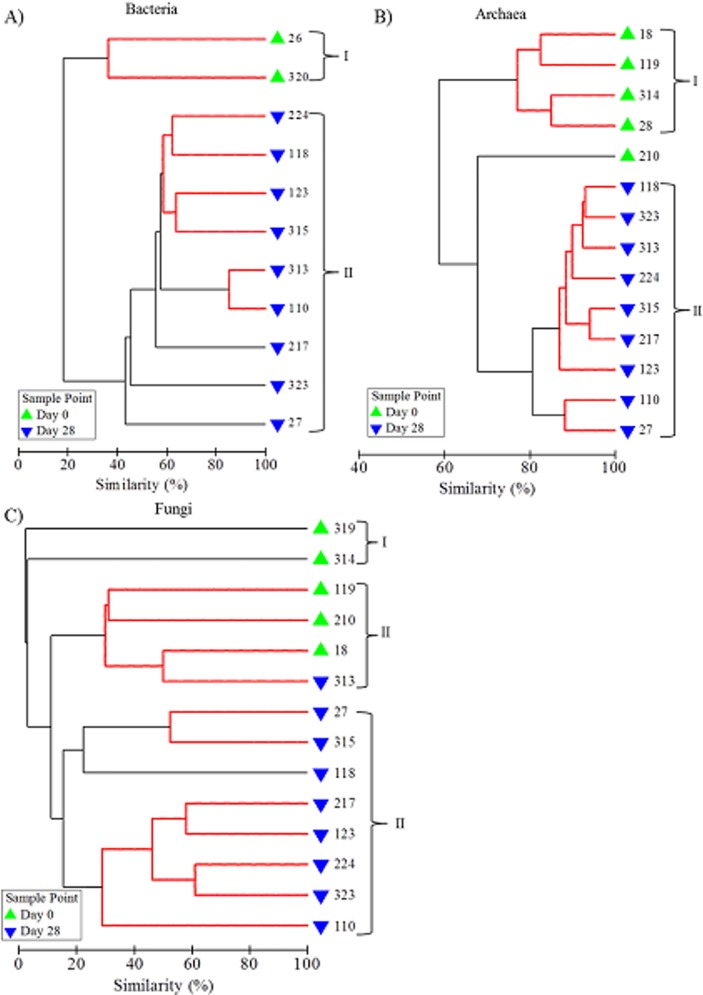
Cluster analysis of similarity using fingerprint profiles to show the similarity between A) bacterial, B) archaeal and C) fungal drinking water biofilm communities. Relative abundance data was derived from T-RFLP or ARISA analysis, sample identification numbers are shown and clusters are indicated with a bracket and number. Red lines indicate profiles not significantly dissimilar according to SIMPROF analysis.

Comparisons of microbial community structure at each sample point demonstrated that, for each microbial group, Day 28 profiles clustered independently from those of the Day 0 biofilms (Fig. 5) and contained significantly different compositions of T-RFs (bacteria, global R = 1.0000, p = 0.0018; archaea, global R = 0.822, p = 0.0005) or ARISA amplicons (global R = 0.593, p = 0.0005). With respect to bacteria and archaea two distinct clusters were observed, with the exception of one replicate (210) in the archaeal community analysis which did not cluster with the communities present in Day 0 biofilm samples (cluster I) or with the biofilm communities present at Day 28 (cluster II, Fig. 5). Fungal community profiles formed three clusters (Fig. 5), the second of which incorporated Day 0 samples and one Day 28 sample (replicate 313). However, when analysed by presence/absence rather than relative abundance, all replicates from Day 0 clustered independently from those from Day 28, suggesting that the same ARISA amplicons were dominant between the samples in cluster II but that there was a difference in the richness of ARISA fragments between the two time points.

Little variation existed between replicates (Table 4) with the greatest variation occurring between fungal community profiles. Nevertheless, profiles were often indistinguishable from at least one other profile from the same sample point, assessed by SIMPROF (indicated by the red lines in Fig. 5). The fungal community profiles were also the most heterogenic between Day 0 and Day 28 biofilms (average similarity 7.35%), followed by the bacteria (18.21%) and archaea (61.56%) communities. Analysis of the T-RFs/ARISA amplicons which accounted for the majority (60%) of the differentiation between the Day 0 and Day 28 biofilm communities demonstrated that some T-RFs or amplicons were unique to Day 0 samples and that 16 bacterial T-RFs, 1 archaeal T-RF and 7 fungal ARISA amplicons were only present at Day 28.

## Discussion

A triple staining, CLSM imaging and novel DIA protocol was established by empirical testing and applied, for the first time, to concurrently visualise and quantify cells, carbohydrates and proteins of biofilms from a full-scale DWDS facility. It is recognised that fluorophores applied to target cells will stain extracellular DNA (eDNA) as well as intracellular DNA, however, eDNA has been reported at very low concentrations in EPS and, if present, is likely to be in concentrations below the limit of detection of staining methods [16]. The above combination of stains was previously used to investigate the EPS of aerobic granules from a bioreactor fed with synthetic wastewater and sludge [36]. However, herein, this fluorophore combination has been applied to characterise microbial biofilms that develop naturally upon the surface of an engineered, chlorinated DWDS system, with an oligotrophic environment contrasting considerably from that of wastewater. A novel advance in the DIA approach has enabled quantitative analysis of each biofilm component, incorporating a method to establish biofilm “thickness” which is unaffected by the uneven scaffold of the internal DWDS pipeline plastic surface and is independent of the potential biases caused by investigator-selection of the Z-stack limits.

Previously, the process of material accumulation at the pipe wall of a DWDS has been referred to as gravity-driven sedimentation of suspended particles, which may subsequently be re-suspended causing water quality issues such as discolouration [37, 38]. Boxall *et al*. [39] suggested an alternative hypothesis, that material accumulates at the pipe wall in “cohesive layers”, adhered via particle-pipe interactions, in layers of different strengths, which detach when the shear stress exceeds that experienced during accumulation; a concept validated by field and laboratory studies [22, 40] and analogous to the formation and detachment of microbial biofilms, more broadly. If gravity-driven sedimentation is the main driver in biological material formation within DWDS, a difference would be expected between biofilms from the invert of the pipeline in comparison to those from the crown. However, there was no significant spatial variation in biofilm physical characteristics or community structure between locations around the internal circumference of the pipe. Where variation between replicates was observed this was attributed to the heterogenic nature of biofilms, and their stochastic development [41]. Therefore, we conclude that the cohesive layer theory better represents the accumulation of biological material within the context of DWDS than gravity-driven sedimentation. This is in contrast to wastewater networks, where material does accumulate to a greater degree on the invert of the pipeline and comprises bacterial communities distinct from those at the crown [42].

At Day 28 the volume of each stained component increased compared to Day 0, as did the EPS-to-cell ratio, indicative of the development of a more mature biofilm, producing an EPS matrix to adhere to the substrata, consistent with previous studies which identified EPS as the main biofilm component [3, 14]. Within a wastewater inoculated biofilm developed at a Reynolds number of 4000, the quantified EPS (carbohydrates only) and cells were shown to increase over 31 days but no significant difference in the EPS-to-cell ratio was reported [12]. It is possible that the switch in dominance of EPS over cells, observed here in biofilms within a chlorinated drinking water system, is due to the need for a greater protection of the cells from a more challenging environment (e.g. oligotrophic, higher disinfectant residuals) than that of the wastewater conditions investigated by Wagner *et al*. [12].

In the present study, the EPS within DWDS biofilms was consistently dominated by carbohydrates; generally, proteins were found in volumes lower than that of the cells. Analysis of the proteins in comparison to carbohydrates within DWDS biofilms is rare but such comparisons exist in biofilms from other environments, such as activated sludge flocs, where proteins were more common than carbohydrates [11] or in *Pseudomonas fluorescens* biofilms cultured within a reactor, in which carbohydrate was the dominate EPS component [14]. Although, in the latter example, the EPS was studied via extraction based methods rather than fluorescent microscopy. Raman microscopy and CLSM analysis of cell/carbohydrate and cell/protein stained biofilms cultured within a wastewater sludge seeded reactor also found carbohydrate to be the most abundant EPS component while proteins could not be detected in biofilms younger than 31 days [12]. In contrast, this study has shown that protein is present in DWDS biofilms at detectable quantities, albeit at lower volumes than cells or carbohydrates. The consistent predominance of carbohydrates reported in the literature, for biofilms from different growth conditions, suggests carbohydrates are the primary EPS component, perhaps as they play a greater role in biofilm structural stability (cohesion and adhesion) than proteins. Möhle *et al*. [43] provide evidence for this theory as biofilms inoculated with activated sludge were found to contain particularly high amounts of carbohydrate in the basal layer, which was found to be more stable than the biofilm at the surface.

Cells, carbohydrates and proteins exhibited a similar spread but were not uniformly distributed throughout the biofilm, nor did the different biofilm components completely co-localise. The results presented herein enabled, for the first time, comparison of the arrangement of cells, carbohydrates and proteins throughout a cross section of a DWDS biofilm, in relation to each other. The differential location of the cells, carbohydrates and proteins was observed, including regions covered solely by cells or one of the EPS molecules, a trend also highlighted by Stewart *et al*. [44], with respect to cells and carbohydrates. While the presence of cell free areas where EPS is observed could be due to the cell volume being below the limit of detection, it seems more likely that these EPS regions represent sites from which cells have migrated, been detached or lysed. The different location of the EPS throughout the three-dimensional structure of the biofilm, and in relation to the cells, has not been extensively considered in the literature. However, within wastewater-fed biofilms a greater cell concentration was reported nearer to the surface in contact with the bulk phase, attributed to a better nutrient and oxygen supply [12] but no analysis of EPS distribution was presented. In contrast, this study has established that the peak location of the EPS within biofilms from a chlorinated DWDS was nearer to the biofilm-water interface than the cells. Potentially, as microorganisms produce EPS, the molecules may be preferentially secreted above the cells to form a barrier protecting against the potentially harmful physico-chemical stresses (e.g. shear forces, disinfectant residual) imposed by the water phase.

In addition to variation in physical structure between Day 0 and Day 28, the biofilms also experienced a change in microbial community structure; the bacterial, archaeal and fungal relative richness increased during the development phase, although the archaeal communities were less complex than the bacterial or fungal communities. Previous drinking water studies have focused upon bacteria [5, 6] or fungi [45]. In contrast, there had been limited research considering archaea in DWDS due in part to the historical difficulties in culturing these microorganisms and that most studies focus solely on one taxonomic domain, typically bacteria. Of those studies which have looked for archaea, some have concluded that they could not be detected [46] while others have confirmed their presence [47]. However, these studies have been based upon samples taken from bench-top systems or taps rather than biofilms from within pipelines, as has been possible with the experimental system presented here. Biofilm studies from environments other than DWDS also found bacterial diversity to be greater than that of archaea [48]. A review of a range of microbial community studies from an array of environments established that reduced archaeal diversity is inherent across various habitats [49]. Archaeal communities may be less diverse than bacteria due to different interactions with the environment, potentially expressing less physiological flexibility. Interestingly, around two thirds of the archaeal libraries (16S rRNA gene) assessed consisted of rare phylotypes, the same proportion as seen in bacterial libraries [49]. This highlights the importance of archaea, which have not been widely investigated, particularly in the drinking water environment where the major focus is bacteria. The identification of several archaeal T-RFs and fungal ARISA amplicons demonstrated in the present study further highlights their role as an important, quantifiable part of the microbial community within DWDS biofilms.

The absence of some amplicons or T-RFs and the presence of others unique to Day 28 biofilms, compared to Day 0, suggests the existence of initial colonising species which were replaced by secondary colonisers. The successional integration of different bacterial species into a biofilm has been stated to be driven, at least in part, by co-aggregation—a process by which cells of different species attach to each other [50]. This phenomenon has previously been observed in laboratory cultivated aquatic biofilms [51] and in bacteria extracted from a drinking water biofilm [52]. In combination with the results presented herein, it seems that bacterial successional colonisation in DWDS is plausible and fungal and archaeal communities may experience this also.

## Conclusion

The novel, full-scale experimental system and analyses presented herein, provide a detailed approach to characterize the structure of drinking water biofilms formed under real-world conditions (hydraulics, physico-chemistry and microbiology). The CLSM and DIA applied are sufficiently sensitive for use in analysing drinking water biofilms and enable characterisation of biofilm physical structure across a variety of parameters. Furthermore this research presents a unique integration of physical (microscopy based) and microbial community structure assessment of DWDS biofilms. Application of the method showed that, within 28 days old biofilms, cells accounted for a smaller proportion of the biofilm than EPS and that the microbial fraction was comprised of bacterial, archaeal and fungal communities. Carbohydrates were the predominant component, although proteins were detected, and the greatest coverage of EPS occurred above that of the cells. Biofilm physical composition or community structure was unaffected by the position around the circumference of the pipe, demonstrating the role of microorganisms in material accumulation within chlorinated DWDS. Overall, this approach has provided a novel insight into the microbial community structure, EPS matrix structure, composition and the architecture of multi-species biofilms. The analysis approach provides an opportunity to investigate the impact of environmental variation upon the structure of biofilms from DWDS and may be applied across an array of engineered systems (e.g. waste water networks, dental waterlines, jet fuel supply lines). Such application will enable new understanding of biofilms, their roles and interactions with the infrastructure and water phase, ultimately aiding biofilm management, which within the context of DWDS will enable the provision of a safe water supply into the future.

## Supporting Information

S1 Fig Schematic representation and analysis of lambda-Z-stack data produced via confocal laser scanning microscopy. A) Schematic of a lambda(λ)-Z-stack comprised of *xyλ* images/slices taken at different focal depths (Z) throughout the sample, with an optical slice depth of 4.7 μm (i.e. slice thickness for which light is collected); B) Detail of the optical interval (2.35 μm) between adjacent slices; C) An example λ-Z-stack gallery showing the determination of an emission signal. Example shown is based on excitation at 633 nm, emission collection over 650.7–704.2 nm, into five bins, 10.7 nm wide; midpoint values of the bins are shown in the lambda and Z dimensions. D) Hypothetical area distributions plots, with either the same volume (V) or “area coverage peak × image area” (P) values; spread values (μm) overlaid, spread calculated using Equation 2 (see text for details).
(TIF)

S2 Fig Auto-fluorescence of the unstained controls, with excitation at 488 nm. A) Example of the unstained plastic and biofilm compared to a FITC stained sample, scale bar 100 μm; B) Seven unstained biofilm emission spectra, imaged using FITC settings (488 nm excitation); Intensity measured in arbitrary units, ROI = region of interest, which refer to the seven FOV.
(TIF)

S1 Table Bulk water quality, based on weekly spot checks. (DOC)

S1 Table Fluorophore combinations tested with the drinking water biofilm samples in this study. (DOC)

S2 Table Final (optimised) Confocal Laser Scanning Microscope settings used to image triple stained drinking water biofilms. (DOC)
