# Supplementary figures and images for: Characterisation of the Physical Composition and Microbial Community Structure of Biofilms within a Model Full-Scale Drinking Water Distribution System
Source: PLoS One. 2015 Feb 23;10(2):e0115824. doi: 10.1371/journal.pone.0115824 (PMC4338064; doi:10.1371/journal.pone.0115824)

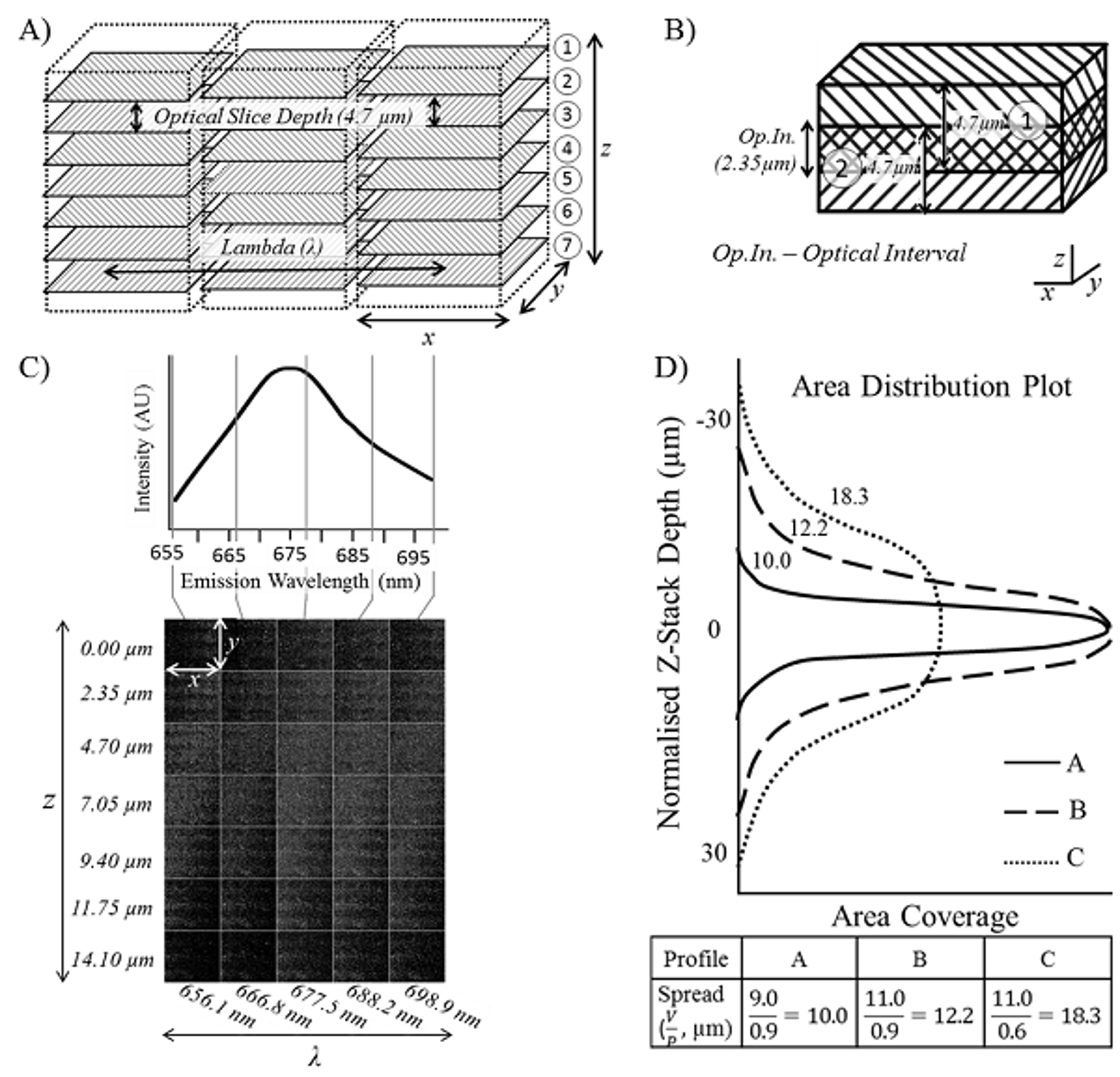

Supplement: S1 Fig — A) Schematic of a lambda(λ)-Z-stack comprised of xyλ images/slices taken at different focal depths (Z) throughout the sample, with an optical slice depth of 4.7 μm (i.e. slice thickness for which light is collected); B) Detail of the optical interval (2.35 μm) between adjacent slices; C) An example λ-Z-stack gallery showing the determination of an emission signal. Example shown is based on excitation at 633 nm, emission collection over 650.7–704.2 nm, into five bins, 10.7 nm wide; midpoint values of the bins are shown in the lambda and Z dimensions. D) Hypothetical area distributions plots, with either the same volume (V) or “area coverage peak × image area” (P) values; spread values (μm) overlaid, spread calculated using Equation 2 (see text for details). (TIF) [file pone.0115824.s001.tif]

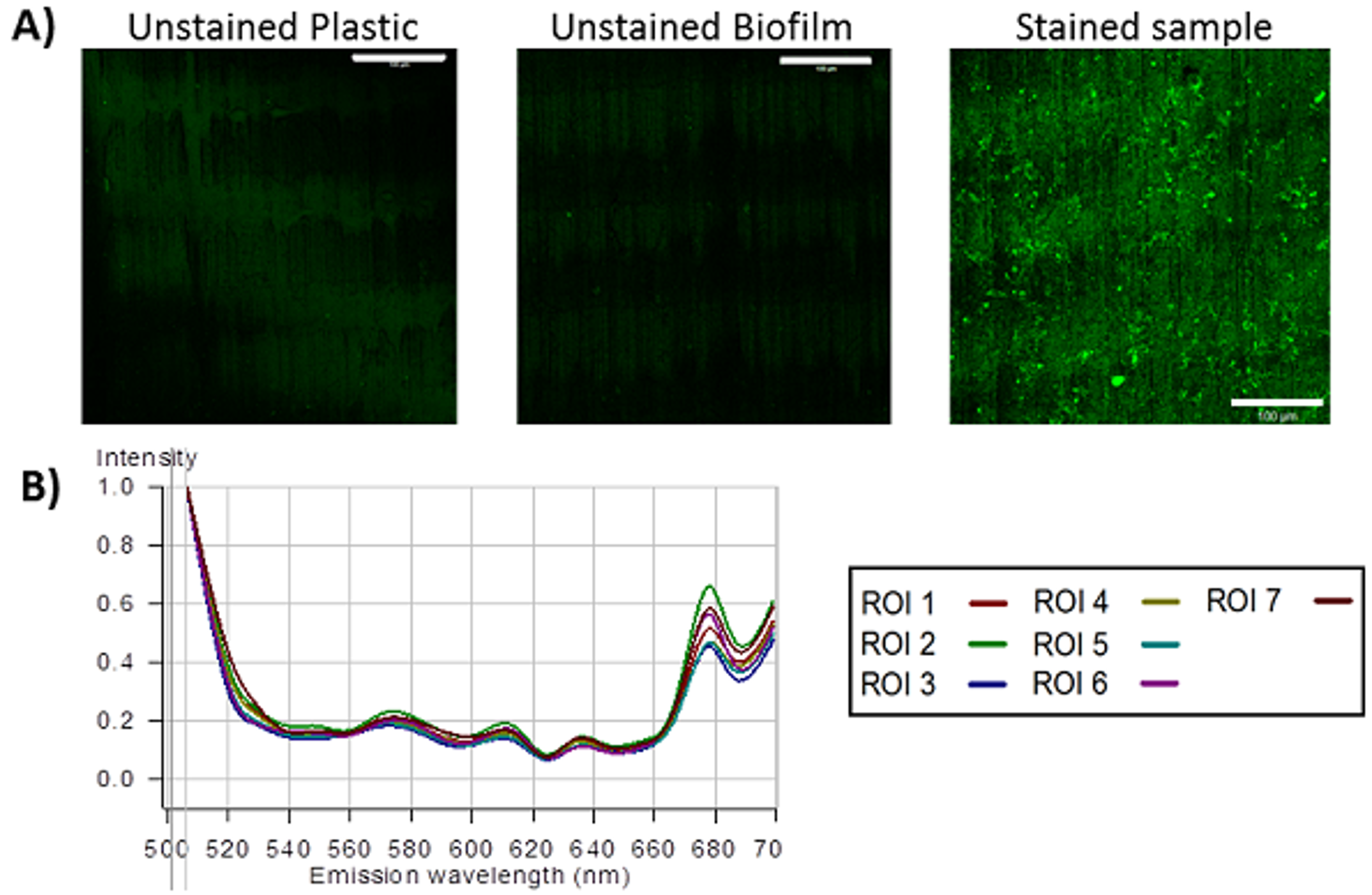

Supplement: S2 Fig — A) Example of the unstained plastic and biofilm compared to a FITC stained sample, scale bar 100 μm; B) Seven unstained biofilm emission spectra, imaged using FITC settings (488 nm excitation); Intensity measured in arbitrary units, ROI = region of interest, which refer to the seven FOV. (TIF) [file pone.0115824.s002.tif]
