# Supplementary material for: Characterisation of the Physical Composition and Microbial Community Structure of Biofilms within a Model Full-Scale Drinking Water Distribution System
Source: PLoS One. 2015 Feb 23;10(2):e0115824. doi: 10.1371/journal.pone.0115824 (PMC4338064; doi:10.1371/journal.pone.0115824)
Supplement: S1 Table — (DOC) [file pone.0115824.s003.doc]

Table S 1 Bulk water quality, based on weekly spot checks.

| **Water Quality Parameter** | **Analysis Method^B^** | **Range (Min – Max)** | **Mean (St.Dev)** | **Median** | **UK Standards ^D^** |
| --- | --- | --- | --- | --- | --- |
| Total Chlorine –Inlet (mg l^-1^) **^A^** | Hanna Chlorine Meter HI96711 | 0.08 - 0.49 | 0.22 (0.20) | 0.11 | Max 5.00 |
| Total Chlorine – Tank (mg l^-1^) |  | 0.00 - 0.47 | 0.23 (0.19) | 0.26 | Max 5.00 |
| Turbidity (NTU) | Hach 2100Q portable Turbidimeter | 0.11 - 0.82 | 0.39 (0.24) | 0.30 | 1.00 – 4.00 ^E^ |
| Iron (µg l^-1^) | ICPOES ^C^ | 15.00 - 24.00 | 19.47 (2.85) | 18.00 | 200.00 |
| Manganese (µg l^-1^) | ICPOES ^C^ | 2.40- 3.10 | 2.70 (0.25) | 2.70 | 50.00 |
| pH | Hanna HI991003 portable multi-probe | 7.00-8.38 | 7.43 (0.54) | 7.07 | 6.50 – 9.50 |
| Oxidising Redox Potential (mV) | Hanna HI991003 portable multi-probe | 221 – 500 | 324 (102.46) | 288 | NONE |
| Temperature – Inlet (°C) | Hanna HI991003 portable multi-probe | 15.1 - 17.7 | 16.1 (1.00) | 15.6 | NONE |
| Temperature – Tank (°C) | Hanna HI991003 portable multi-probe | 15.6 - 17.6 | 16.4 (0.62) | 16.3 | NONE |

^A^ n=9, data only for Day 7-Day 21; all other parameters n=15; ^B^ All equipment was calibrated and maintained in accordance with the manufacturers’ guidelines; ^C^ Inductively Coupled Plasma Optical Emission Spectroscopy; samples analysed by AlControl Laboratories, Rotherham, UK; ^D^ Standards in place in the UK based on Drinking Water Inspectorate (DWI) or European Union (EU) legislation, or in the case of chlorine the World Health Organisation (WHO) as DWI and EU do not provide a standard; ^E^ Max values, water leaving a treatment plant must be ≤ 1 NTU, end point water ≤ 4 NTU. N.B. Min = minimum, Max = maximum, St.Dev = standard deviation.
