# Supplementary material for: Characterisation of the Physical Composition and Microbial Community Structure of Biofilms within a Model Full-Scale Drinking Water Distribution System
Source: PLoS One. 2015 Feb 23;10(2):e0115824. doi: 10.1371/journal.pone.0115824 (PMC4338064; doi:10.1371/journal.pone.0115824)
Supplement: S2 Table — (DOC) [file pone.0115824.s005.doc]

Table S 1 Fluorophore combinations tested with the drinking water biofilm samples in this study.

| **Target Biofilm Components** | **Combination Reference №** | **Fluorophore Combination ^A^** | **Successful Separation ^B^** |
| --- | --- | --- | --- |
| Cells (nucleic acids) and Carbohydrates | 1 | SYTO 9 and Con A Rho | 🗸 |
|  | 2 | SYTO 9 and Alexa Fluor 647 | 🗸 |
|  | 3 | BacLight Live-Dead and Con A Rho | X |
|  | 4 | SYTO 63 and Con A Rho | 🗸 |
|  | 5 | SYTO 63 and Alexa Fluor 647 | X ^C^ |
| Cells and Proteins | 6 | SYTO 9 and SYPRO Orange | X |
|  | 7 | SYTO 9 and FITC | X ^C^ |
|  | 8 | SYTO 63 and FITC | 🗸 |
| Proteins and Carbohydrates | 9 | SYPRO Orange and Con A Rho | X |
|  | 10 | SYPRO Orange and Alexa Fluor 647 | 🗸 |
|  | 11 | FITC and Con A Rho | 🗸 |
| Cells, Proteins and Carbohydrates | 12 | SYTO 63, FITC and Con A Rho | 🗸 |

^A^ See Table 1 for excitation and emission details of each individual fluorophore; ^B^ 🗸 indicates emission spectra of the two fluorophores could be resolved, X indicates spectra could not be resolved; ^C^ Eliminated based on theoretical incompatible excitation and emission spectra rather than empirical evidence.
